# Prognostic Impact of Serial Imaging in Severe Acute Respiratory Distress Syndrome on the Extracorporeal Membrane Oxygenation

**DOI:** 10.3390/jcm12196367

**Published:** 2023-10-05

**Authors:** Martin Balik, Michal Maly, Michal Huptych, Masego Candy Mokotedi, Lukas Lambert

**Affiliations:** 1 Department of Anesthesiology and Intensive Care, 1st Faculty of Medicine, Charles University and General University Hospital, 128 08 Prague, Czech Republic; michal.maly@vfn.cz; 2 Czech Institute of Informatics, Robotics and Cybernetics (CIIRC), Czech Technical University, 190 00 Prague, Czech Republic; michal.huptych@cvut.cz; 3 Department of Radiodiagnostics and Interventional Radiology, Institute of Clinical and Experimental Medicine, 140 00 Prague, Czech Republic; mudrmokotedi@gmail.com; 4 Department of Radiology, 1st Faculty of Medicine, Charles University and General University Hospital, 128 08 Prague, Czech Republic

**Keywords:** chest ultrasonography, chest X-ray, chest computer tomography, acute respiratory distress syndrome, extracorporeal membrane oxygenation, COVID-19 pneumonia, pneumothorax, barotrauma

## Abstract

Background: The impact of serial imaging on the outcome of ICU patients has not been studied specifically in patients with high illness severity. Methods: The authors sought a relationship between the numbers of antero-posterior supine chest X-rays (CXR), computed tomography (CT) examinations, and outcome in a cohort of 292 patients with severe COVID-19 ARDS collected over 24 months in a high-volume ECMO center with established ultrasound and echocardiographic diagnostics. Of the patients, 172 (59%) were obese or morbidly obese, and 119 (41%) were treated with ECMO. Results: The median number of CXRs was eight per 14 days of the length of stay in the ICU. The CXR rate was not related to ICU survival (*p* = 0.37). Patients required CT scanning in 26.5% of cases, with no relationship to the outcome except for the better ICU survival of the ECMO patients without a need for a CT scan (*p* = 0.01). The odds ratio for survival associated with ordering a CT scan in an ECMO patient was 0.48, *p* = 0.01. The calculated savings for not routinely requesting a whole-body CT scan in every patient were 98.685 EUR/24 months. Conclusions: Serial imaging does not impact the survival rates of patients with severe ARDS. Extracorporeal membrane oxygenation patients who did not need CT scanning had significantly better ICU outcomes.

## 1. Introduction

Imaging methods are a mainstay in the diagnosis of severe respiratory failure in the critically ill. Imaging helps guide hemodynamic management, therapy of respiratory insufficiency, and the strategy of mechanical ventilation [1,2,3,4,5,6,7]. In addition to imaging methods such as portable chest X-ray (CXR), ultrasound, and echocardiography, computed tomography (CT)is often supplemented. Its impact on management is crucial, yet it is also associated with a burden of transport to the radiology suite, medical radiation, contrast agent administration, and additional costs [8,9]. 

The impact of routine daily imaging on intensive care unit (ICU) patients is also controversial because the indications for imaging methods have not been stratified according to illness severity [10]. A mechanically ventilated, primarily comatose patient with absent lung pathology may not benefit from serial chest imaging compared to an aggressively ventilated patient with severe ARDS.

The expansion of ultrasound methods in the intensive care setting may influence the need for radiographic imaging. Increased use of echocardiography, chest, and point-of-care ultrasound (POC-US), including focused-ultrasonography-in-trauma protocol (FAST), may reduce the need for radiographic methods even in the critically ill with high illness severity [11,12,13,14,15]. 

However, recent recommendations to pursue routine serial CT scanning of all patients with severe cardiorespiratory failure, especially those on ECMO, are not supported by sufficient evidence [16,17,18]. A potential benefit of early CT scanning has been suggested for patients after an out-of-hospital cardiac arrest (OHCA) rescued by extracorporeal membrane oxygenation (ECMO) in the form of extracorporeal cardiopulmonary resuscitation (e-CPR) [19,20,21]. Regarding the high prevalence of resuscitation-associated trauma, whole-body CT scanning may be logically justified, especially in cases with inconclusive results in an initial POC-US and FAST ultrasonographic exam [22,23]. The significant prevalence of rib and sternal fractures (11–79%), pneumo/haemothorax (9–32%), lung contusions, and infiltrates (20–91%) on initial thoracic CT may evade diagnosis on ultrasound. Moreover, the limited sensitivity of abdominal ultrasound to detect and quantify resuscitation-associated abdominal trauma (especially to the liver, spleen, and gut) makes whole-body CT scans indispensable in inconclusive ultrasonographic examinations [23]. Head CT may reveal brain edema (26–29%), especially if delayed beyond 48–72 h, or intracranial bleeding (4.3–10.7%). A whole-body CT scan post-e-CPR led to intervention in 54%, including 19.4% of patients transitioning to paliative therapy [19,20,21]. Numerous non-cardiac causes of cardiac arrest can be conveniently diagnosed by a CT scan [24]. A study confirming a relationship between routine CT scanning after e-CPR and the outcome is not available. In the large trial on e-CPR, which included patients primarily assessed by echocardiography and ultrasound prior to emergency cannulation in the cath lab, post-admission CT scans were ordered in 39% of cases [25]. A CT scan of patients on the veno-arterial ECMO has its inherent limitations due to the need to manipulate blood flow in contrast agent application and imaging subtraction. The contrast is drawn from the central vein into the ECMO circuit, resulting in dilution and delayed imaging; hence, the quality of imaging depends often on the cooperation between the radiologist and intensivist [26].

A study analyzing the potential impact of serial CT/CXR imaging on the outcome of patients with severe ARDS treated with ECMO is also not available. Radiographic chest imaging facilitates the optimum modality of mechanical ventilation, enabling a patient-tailored approach and having a positive impact on the outcome data [6]. Patients with high illness severity may benefit from serial bedside imaging when on an aggressive mode of mechanical ventilation with a risk of barotrauma. Nonetheless, with growing applications of bedside ultrasound, the rationale for routine and serial CT scanning might be more difficult to justify. A significant survey among German ECMO units showed 56% of patients had whole-body CT scans, 60% required CT of the thorax, 32% had an abdominal CT scan, and 24% were ordered a cranial CT scan [17]. The number of detected pathologies was related to a worse outcome, especially the CT head pathologies (17%), which significantly changed patient management [16,18]. The intracerebral bleeds related to anticoagulation on ECMO are worrisome and often outcome-changing complications, which highlights the indispensable role of cranial CT scanning [27]. 

In this study, we sought a relationship between the number of bedside serial antero-posterior supine CXRs, CT scans, and the ICU outcome in an existing large cohort of severe COVID-19 ARDS patients treated with ECMO [28]. The radiographic imaging was not ordered routinely on a daily basis (CXR) or at regular intervals to monitor the course of the ARDS (CT). All imaging was diagnostic or periprocedural. The hypothesis was that both CXR and CT numbers might not relate to an ICU outcome, including in obese patients and patients on ECMO. The authors hypothesized that if fewer studies were performed, then important clinical information might have been missed. In contrast, more frequent radiographic imaging might have been associated with a better ICU outcome in aggressively ventilated patients. An exploratory analysis of an already existing and published dataset [28] was performed in a high-volume ECMO center with established ultrasound and echocardiographic diagnostics of critically ill patients. 

## 2. Materials and Methods

A retrospective analysis of COVID-19 patients with severe ARDS according to the Berlin 2011 criteria [29] admitted to a single high-volume ECMO center between March 2020 and March 2022 was performed. The authors sought to determine the impact of the number of antero-posterior CXRs and CT scans on the resultant ICU outcome. The requirements for imaging were stratified according to the body mass index (BMI), i.e., obese (BMI > 30) and non-obese (BMI ≤ 30) patients, and patients managed with and without ECMO. The exclusion criteria included all patients with mild ARDS and/or the absence of a weighted bed for BMI measurement. The data was retrieved from the hospital information system and included demographic characteristics, patients’ histories, clinical data—body mass index (BMI), initial status at ICU admission, and severity scores [Acute Physiology and Chronic Health Evaluation (APACHE IV), Sequential Organ Function (SOFA)]. Imaging examinations were retrieved from an existing and already published dataset [28]. 

Since 2009, the Prague General University Hospital’s ECMO center treats approximately 120 ECMO patients annually, with the numbers largely increasing during the 2020–22 COVID-19 pandemic. The department is a pioneer in the field of critical care echocardiography and ultrasonography. Extracorporeal life support (ECLS) patients are entered into the extracorporeal life support organization (ELSO) database, and the intensive care or interventional cardiology teams perform 80% of all emergency ECMO cannulations. The ECMO center also provides an emergency service for cardiorespiratory failure for the whole country in addition to an e-CPR service for the Prague metropolitan area. 

### Statistical Analysis

After verifying the distribution of data, the continuous data is expressed as the median and interquartile range (IQR), and differences between the groups were tested with the Mann-Whitney U test. The categorical data is expressed as the number of probands and percentage of a given group and evaluated using the Fischer’s exact test. The Rank correlation with Spearman’s coefficient is utilized for correlation analysis of the number of RTG records with the length of stay and outcome in the ICU. The design of the regression analysis followed the original method published previously [28], now with added data on imaging. The linear regression analysis and the Mantel-Haenszel test were performed in order to determine the odds ratio of a particular complication and its relationship to the frequency of radiographic imaging. Cox proportional-hazards regression analysis was employed to test the various risk factors and their relationships to the outcomes. A *p*-value below 0.05 was considered significant. 

## 3. Results

### 3.1. Chest X-ray

For a period of 24 months between March 2020 and March 2022, a total of 292 patients with severe ARDS were included in the analysis. Their median age was 57 years (IQR 48–69), with men comprising a total of 194 (66.4%). Out of the 292 patients, 173 were treated conservatively, and 119 (40.8%) were treated with ECMO. The characteristics of the patients relevant to chest imaging, including the outcome data, are summarized in Table 1. The patients were divided into the obese group (*n* = 171, 58.6%) and the non-obese group (*n* = 121, 41.4%). 

The indications for serial CXRs were diagnosis of the respiratory apparatus (45%) and periprocedural related, for example, to central line or chest drain placement (55%). The median number of CXRs per patient was eight (IQR 4–14) across all classes of the body mass index. The univariate Mann–Whitney test did not find a significant relationship between the number of CXRs and ICU outcome, which was relevant for both obese (BMI > 30) and non-obese (BMI ≤ 30) patients (Table 2). 

**Figure 1:**
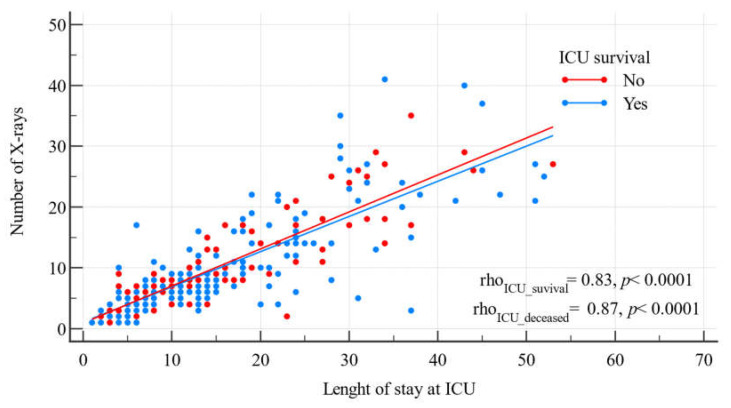
A scatter diagram showing the relationship between the number of chest X-rays and the length of stay in the ICU. Blue line for ICU deceased, red line for ICU survival.

The number of CXRs strongly correlated with the length of stay of both the survivors and the deceased in the intensive care unit (r = 0.87, *p* < 0.0001, r = 0.83, *p* < 0.0001, respectively, Figure 1).

The relationship between the length of stay and ICU survival was not significant (*p* = 0.54). Furthermore, to eliminate an effect of the length of stay in the ICU, the mean number of CXRs per day in the intensive care unit was analyzed for a relationship to outcome. The mean CXR number per day in the ICU was 0.7 ± 0.32 and showed no significant relationship to the ICU outcome (*p* = 0.37). The odds ratio (OR) for the number of CXRs per day in the ICU for the ICU outcome was 1.2 (0.57–2.49; *p* = 0.63). In addition, we analyzed the multivariable combination of selected clinical factors and the mean number of CXRs per day in the ICU by multivariable logistic regression (Table 3). The relationship was not significant (*p* = 0.45). 

### 3.2. Computed Tomography

The median number of CT scans per patient was zero (IQR 0–1) because only 26.5% of patients with a BMI of 18–40 had at least one CT scan per ICU stay. A total of 145 CT scans were ordered for 77 patients with severe ARDS from the entire cohort of 291 patients. The rates of CT scanning were 26–33% across the classes of BMI, except for the morbidly obese patients (BMI > 40) who required a CT scan in 14% of the cases. The highest BMI of a patient with a CT scan was 44.2. The median number of CT scans in those who had at least one CT scan was one (IQR 1–2). 

The indications for CT scans were mainly CT of the brain for a suspected intracranial pathology (74 scans), CT angiography indicated typically for a suspected pulmonary embolism (50 scans), and CT of the trunk obtained mostly to search for the source of sepsis or abdominal pathology (21 scans). 

The relationship between the number of CT scans and the ICU outcome was evaluated with respect to the BMI (18–44.2) and ECMO therapy. The highest BMI with a CT scan in our study was 44.2. The study did not find any differences in outcomes related to the presence of a CT scan performed, ECMO therapy, or the length of stay in the ICU (Table 4). 

The ICU outcome of all patients treated with ECMO who required a CT scan (only patients with BMI < 45) was not different (49% rates of ECMO in the surviving vs. 60% in the deceased, *p* = 0.46). The absence of a CT scan in patients on ECMO, however, predicted the prognosis of patients (29.1% in survivors vs. 47.4% in deceased in the ICU, *p* = 0.01). The OR for survival associated with ordering a CT scan (Cox analysis) for ECMO patients was 0.48, *p* = 0.01. 

**Figure 2:**
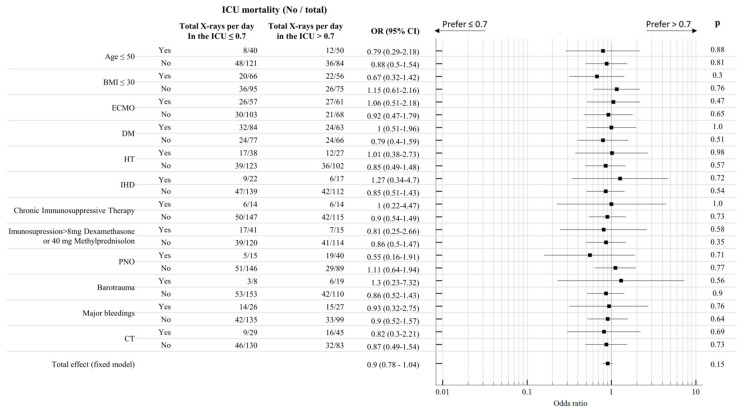
Mantel–Haenszel analysis of factors tested for their relationship to the adverse outcome (death in the ICU)—the mean X-ray number per day in the ICU and ICU outcome. The total effect of 0.9 represents the impact of selected variables on the impact of imaging on a patient’s outcome (BMI body mass index, DM diabetes mellitus, HT hypertension, IHD chronic intermittent hemodialysis, and PNO pneumothorax).

To evaluate the impact of selected diagnostic and therapeutic factors in relation to the purchased CXRs, CT scans, and ICU outcome, we performed the Mantel-Haenszel analysis, which calculated the odds ratio for the presence of a particular complication. For the analysis, we used the mean CXR number per day in the ICU as a cut-off value (Figure 2). The factor of the CT scan was shown to be not statistically significant in relation to the length of the stay in the ICU and an adverse outcome. The Mantel-Haenszel analysis showed a total effect of the various selected clinical factors on the impact of less or more frequent radiographic imaging close to one (0.9, *p* = 0.15), which was not significant. 

Financial analysis of our data calculated an average cost of EUR 459 for a whole-body CT scan and EUR 304 for a head or chest CT. With CT scan rates in our cohort being 27%, the estimated saving on 292 severe ARDS patients was EUR 98.685 if a CT had been ordered in all 100% cases over two years, compared to only 27% of the patients in whom the CT scans were indicated. 

## 4. Discussion

Patients with severe ARDS treated with ECMO in an ICU with an established use of ultrasound required CT scanning in 26.5% of cases. The median number of CXRs was eight per the median 14-day length of stay in the ICU, which is a mean of 0.7 CXR per day of the ICU stay. The rate of CXR lower or higher than the median of eight was not related to better survival, therefore suggesting there is no relationship to the ICU outcome of the aggressively ventilated patients. CT scanning did not show a relationship to survival; however, it showed a better ICU outcome in ECMO patients who did not need a CT scan. In other words, patients with the least requirement for CT scanning were likely to have a more favorable course of the disease, therefore attracting fewer disease-related complications and thus having fewer indications for a CT. The adverse events during the ICU stay were likely related to the number of complications requiring radiographic methods. The Mantel-Haenszel analysis showed that the total effect of various clinical factors on the impact of less or more frequent radiographic imaging was close to one, that is, not significant. Interestingly, this also includes mediastinal barotrauma and pneumothorax, which were routinely and primarily managed by chest ultrasound in the absolute majority of the included critically ill patients. 

The idea of ordering daily CXR in critically ill patients is not novel; however, our study adds information on the potential benefit of routine bedside CXR in aggressively ventilated patients with severe cardiorespiratory failure. Considering the ventilator settings (Table 1) and barotrauma rates of up to 33%, this approach seems to be fully justified. Importantly, 2/3 of the patients were obese, and 1/5 were morbidly obese [28], increasing the side effects and risks of mechanical ventilation. The daily radiology ward rounds may include direct ventilation therapy [5,6,7,30], further ultrasound scanning [13,14], and increased patient safety. Albeit retrospective, this study shows that, besides some primary indications, CT scanning can be reserved for ICU-related complications, and, as a result, its absence in patients on ECMO was associated with a better outcome. 

A combination of chest ultrasound, echocardiography, and chest X-rays was apparently sufficient for up to 3/4 of patients with severe ARDS, avoiding the need for a CT scan. The CT scan exposes patients to ionizing radiation and potential side effects brought on by the administration of contrast agents used to enhance imaging. The CT scanning approach can have negative implications due to the transfer of the ICU patients to the radiology suite, which is not always safe in severe illnesses with the risk of instability associated with transport [8,9,18,31]. There is also an increased transport-associated workload for the ICU staff, which may be a sensitive issue at times of shortage. Epidemiology issues and the transmission of contagious diseases, e.g., COVID-19, are important factors with implications for an out-of-ICU environment where disinfection and cleaning are required in the CT suite, which may interrupt routine work. 

Not negligible is the financial impact of imaging on the payment-per-diagnosis reimbursement of care, which may differ from a generous system of proportional payment for care. In the former system, imaging costs are reimbursed from a lump sum assigned to a diagnosis. With the rates of CT in our cohort being around 27%, the estimated savings on 292 severe ARDS patients were up to EUR 100.000 if a CT had been ordered in 100% of cases over the two years. 

The study has multiple limitations. With regards to the high numbers of patients discharged to local hospitals, we did not track the imaging methods ordered after a discharge from the ICU. Therefore, we cannot reliably provide information on hospital outcomes due to a lack of data beyond discharge from the intensive care unit. The nature of the retrospective post-hoc analysis of an already published data set gives insight into the proceedings at a high-volume ECMO center with an established system of bedside imaging, namely the ultrasound methods. The extreme prevalence of obesity and morbid obesity among the COVID-19 patients likely had an impact on access to CT scanning, with some weight limitations on the method. To generalize the findings of the study, we would need to randomize patients into groups with routine, frequent serial radiographic imaging compared to a group with the implementation of our proposed approach based on a combination of primary ultrasound methods with subsequent X-ray or CT imaging. The comparison should include an evaluation of the impacts of these two approaches on the whole system of intensive care and radiology services in the involved institutions.

## 5. Conclusions

The study did not find a relationship between the ICU survival of patients with severe ARDS and radiographic imaging methods. ECMO patients without the need for a CT scan had a significantly better ICU outcome.

## Figures and Tables

**Table 1:** Characteristics of the patients.

	BMI > 30 (*n* = 171)	BMI ≤ 30 (*n* = 121)	*p*-Value
Age (years)	56 (48–65)	61 (51–68)	**0.013**
Weight (kg)	110 (100–120)	82 (75–90)	**<0.001**
Height (m)	1.74 (1.68–1.8)	1.77 (1.7–1.8)	**0.024**
BMI	35.1 (32.1–40.1)	26.3 (24.8–27.8)	**<0.001**
Gender (males)	63.7% (109)	70.2% (85)	0.246
APACHE IV	87 (77–100)	90 (77–100)	0.371
SOFA	10 (8–12)	11 (8–12)	0.480
PaO_2_/FiO_2_ (at admission)	75 (62–101)	75.5 (60–105)	0.883
Orotracheal intubation on admission	95.9% (164)	94.2% (114)	0.505
NIV/HFNO (days)	2 (1–4)	2 (0–5)	0.702
Tracheostomy	75.4% (129)	69.4% (84)	0.254
MV parameters (at admission)			
PEEP	12 (10–14)	11 (8–14)	**0.048**
driving pressure	18 (15–20)	16 (12–18)	**<0.001**
plateau pressure	30 (26–34)	28 (24–30)	**0.002**
Prone position	62% (106)	59.5% (72)	0.623
VV ECMO	45% (77)	34.7% (42)	0.087
Pneumothorax	18.1% (31)	19.8% (24)	0.749
Pneumothorax in ECMO patients	26% (20)	33.3% (14)	0.404
Pneumomediastinum	8.8% (15)	9.9% (12)	0.763
Pneumomediastinum in ECMO patients	18.2% (14)	19% (8)	1.00
ICU LOS (days)	13 (7–22)	13 (6–20)	0.432
Hospital LOS (days)	27 (14–51)	27 (13–66)	0.639
ICU mortality	36.8% (63)	33.9% (41)	0.578
Hospital mortality	49.7% (85)	48.8% (59)	0.873
90-day mortality	48.5% (83)	45.5% (55)	0.603

APACHE, acute physiology and chronic health evaluation; SOFA, sequential organ failure assessment; CAD, coronary artery disease; COPD, chronic obstructive pulmonary disease; HFNO, high-flow nasal oxygen; NIV, non-invasive ventilation; MV, mechanical ventilation; PEEP, positive end-expiratory pressure. Table 1: Comparison of the obese versus non-obese patients (numbers and percentages or medians with interquartile ranges, compared with the Mann–Whitney U or the Fischer´s exact tests). The significant *p*-values below 0.05 are in bold.

**Table 2:** Comparison between the number of chest X-rays (median, IQR) of surviving and deceased patients in the ICU.

Parameter	Surviving (Median, IQR)	Dead (Median, IQR)	*p*-Value
ICU outcome	7 (4; 14), *n* = 187	8 (5; 15.5), *n* = 104	0.23
ICU outcome BMI > 30	8 (5; 14.8), *n* = 107	8 (5; 13), *n* = 62	0.69
ICU outcome BMI ≤ 30	7 (3.5; 12), *n* = 80	8.5 (5; 17), *n* = 42	0.21

**Table 3:** Multivariable logistic regression for selected complications, adverse outcome, and mean number of X-rays per day in the ICU (BMI body mass index, DM diabetes mellitus, HT hypertension, and IHD chronic intermittent hemodialysis). The significant *p*-values below 0.05 are in bold.

Variable	Odds Ratio	95% CI	*p* Value
Age > 50	2.59	1.22–5.09	**0.006**
Gender male	1.25	0.7–2.22	0.44
BMI > 30	1.16	0.64–2.08	0.63
ECMO yes	2.11	1.13–3.94	**0.02**
DM yes	1.39	0.73–2.62	0.32
HT yes	0.96	0.54–1.69	0.88
IHD yes	1.18	0.53–2.64	0.68
Chronic immunosuppressive therapy yes	1.71	0.69–4.21	0.25
Imunosupression > 8 mg Dexamethasone or 40 mg Methylprednisolone yes	1.31	0.67–2.56	0.43
Pneumothorax	1.26	0.63–2.51	0.51
Barotrauma yes	0.42	0.16–1.12	0.08
Major bleeding event yes	2.1	1.01–4.37	**0.046**
Number of X-rays per day in the ICU	1.35	0.61–2.98	0.45

**Table 4:** Comparison of rates of CT scans (percentage, numbers) between survivors and deceased in the ICU.

Parameter	% CT among Surviving (No. of Probands)	% CT among Dead (No. of Probands)	*p*-Value
ICU outcome	29% (49)	25.8% (25)	0.67
ICU outcome ECMO	42.9% (24)	30.6% (15)	0.23
ICU outcome without ECMO	22.3% (25)	20.8% (10)	1.0
ICU LOS (days)	12 (6–19)	12 (8–20)	0.45

## Data Availability

The anonymous study dataset is available upon reasonable request to the authors.
